# Flowers, Ferns, and Ward Decorations

**Published:** 1886-10-09

**Authors:** 


### Flowers, Ferns, and Ward Decorations.

Communications for this column should be addressed, "Gardener," care of the Editor of THE HOSPITAL.

### The History and Mission of the Flowers.

Gardens. "God Almighty first planted a garden, and, indeed, it is the first of all human pleasures," says Bacon, and he might have added "the last" also, for I take it there is no delight on the face of the earth that begins so soon and remains with us so long as this same love of a garden, with its ever-varying succession of sweet scents and glorious colours. Every creature who claims superiority to the beasts that perish must have a place in his heart for flowers, searching with reverent eyes for the meaning of the many messages they hold in dewy cup, 'twixt tender leaf and petal. They are, between earth and heaven, links of spotless fairness, of significance the deepest, passing by, along the season's pathway, a winsome phalanx of unbroken beauty, from January's first pale bud, to the red and white of mistletoe and holly greeting our eyes at Christmastide—eyes that haply may look back through long years to a time when Christmas Day was a day of simple joy and thanksgiving, marking no space that was blank, no vacant chairs or cradle.

Flowers in everyday Life. There are few events in mortal life unconnected with flowers, from the day when we dangle our first daisy-chain, to the hour, far or near, when the season comes, maybe with anguish and bitter weeping, to lay beside our stiffened figures a spray of white and green, to go away with us and stay beside us in the silence of an "everlasting rest"—a little tender gift, the last and most useless, wet with the tears of one who fain would lie where the blossoms lie, following gladly even into the darkness of the grave itself. And long ere this, in the space of time allotted to each one of us, have not flowers been to us, in their delicate meaning, their tender uses, something no other influence would impart, in the early upspringing of youthful hope,—when hope passed into certainty, into the fulfilment of love's completeness, were they not near us, playing their part in the drama of our days, in sunshine and shadow alike, when comedy and tragedy, always so close together, jostled one another on the platform of our existence?

An Unconscious Ambassador. Few there be amongst us to whom, sometime or other, flowers have lent no aid, from Betty and John in the lanes on Sunday afternoon, ogling each other from behind comfortable bunches of sweetwilliam and sothernwood—Betty and John do not talk much—to the first Lady in the land, who perforce had recourse to the medium of flowers ere she could embolden the man of her heart to lay his own at the feet of a Queen. The unconscious ambassador went forth upon its mission and secured for its mistress the possession of a crown and empire more glorious and invaluable than any to be found in the regalia of Great Britain and her mighty dependencies—a crown wrought and engraved with the love of husband and children, an intangible, fair kingdom, where a woman's lips might speak such things as are dear to a woman's heart, when state and pageantry of public life could be laid aside—and all this for the length of twenty years, ere the end which seemed to come so soon. Somewhere hidden away, the silent ambassador, whether rose or lily it matters not, may be lying still, folded out of sight beyond reach of desecrating hands, in the unbroken solemnity bestowed on dead and holy things, known only to one who may look sometimes, with yearning and the bitterness of absolute regret, upon the shrivelled imperfection, and reinvest its withered nothingness with all the glory it possessed over forty years ago—for the idyl it belonged to is over, over and done with a quarter of a century since. One winter night the voice of mourning was heard throughout the land, bells rang muffled peals and the heart of England ached, since the Queen was widowed, and Albert the Good went forth to meet his God, to resign that which one who knew has called "the white flower of an unstained life."

*(To be continued.)*

